# Copper-catalyzed asymmetric conjugate addition of organometallic reagents to extended Michael acceptors

**DOI:** 10.3762/bjoc.11.263

**Published:** 2015-12-03

**Authors:** Thibault E Schmid, Sammy Drissi-Amraoui, Christophe Crévisy, Olivier Baslé, Marc Mauduit

**Affiliations:** 1 Ecole Nationale Supérieure de Chimie de Rennes, UMR CNRS 6226, 11 Allée de Beaulieu, 35708, Rennes cedex 7, France; 2 Institut Charles Gerhardt Montpellier, UMR 5253 CNRS-UM-ENSCM, Ecole Nationale Supérieure de Chimie, 8 Rue de l’Ecole Normale, 34296 Montpellier Cedex 5, France

**Keywords:** conjugate additions, electron-deficient alkenes, enantioselective catalysis, extended Michael acceptors, organometallic nucleophiles, sequential addition

## Abstract

The copper-catalyzed asymmetric conjugate addition (ACA) of nucleophiles onto polyenic Michael acceptors represents an attractive and powerful methodology for the synthesis of relevant chiral molecules, as it enables in a straightforward manner the sequential generation of two or more stereogenic centers. In the last decade, various chiral copper-based catalysts were evaluated in combination with different nucleophiles and Michael acceptors, and have unambiguously demonstrated their usefulness in the control of the regio- and enantioselectivity of the addition. The aim of this review is to report recent breakthroughs achieved in this challenging field.

## Introduction

Amongst the variety of synthetic methods available for the formation of C–C or C–heteroatom bonds, the asymmetric conjugate addition (ACA) of nucleophiles to electron-deficient alkenes is one of the most relevant and versatile for the synthesis of complex chiral molecules [1]. Notably, the design and study of novel families of chiral enantiopure ligands has enabled a fine control of the regio- and enantioselectivity of the reaction, using a variety of nucleophilic and electrophilic substrate associations, with remarkable applications in total syntheses [2].

**Figure 1:**
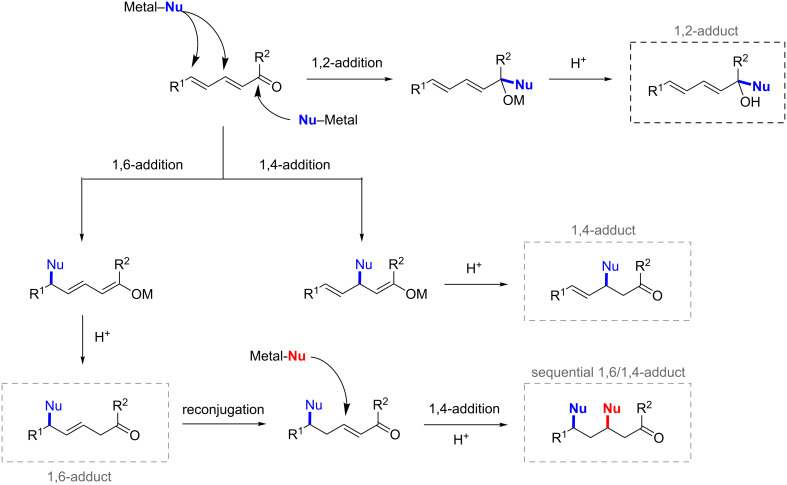
Possible reaction pathways in conjugate additions of nucleophiles on extended Michael acceptors.

Polyenic electron-deficient alkenes are Michael acceptors of high synthetic interest. Indeed, they can undergo successive nucleophilic additions and therefore enable the generation of several new chiral centers [3]. On the other hand, the main challenge associated with polyenic Michael acceptors lies within the regiocontrol of the nucleophilic attack, which can occur at three different positions, at least. The regioselectivity outcome of the ACA reaction depends on many parameters, notably the metal/chiral ligand combination, the structure of the electrophile and the nature of the nucleophile. Figure 1 depicts the various scenarios that can be expected with an α,β,γ,δ-unsaturated Michael acceptor.

Amongst the variety of transition-metal-based catalytic systems that have been evaluated in ACA reactions on extended Michael acceptors [1,3], copper-based systems have been the subject of tremendous interest, which provided dramatic breakthroughs during the last two decades. This review aims to describe the early examples and recent advances in copper-catalyzed asymmetric conjugate additions of organometallic reagents to extended Michael acceptors. First, seminal reports dealing with the reactivity of extended Michael acceptors with regards to copper-based nucleophiles in stoichiometric reactions will be presented. Based on these results, research groups gained better understanding of the origin of the regioselectivity in such processes, and started to develop modern enantioselective catalytic systems. These works will be classified according to the selectivity of the addition (1,6, 1,4 then 1,8 and 1,10), while taking into account the nature of the nucleophile (dialkylzinc, Grignard or trialkylaluminium reagents).

## Review

### Background – first studies

**Figure 2:**
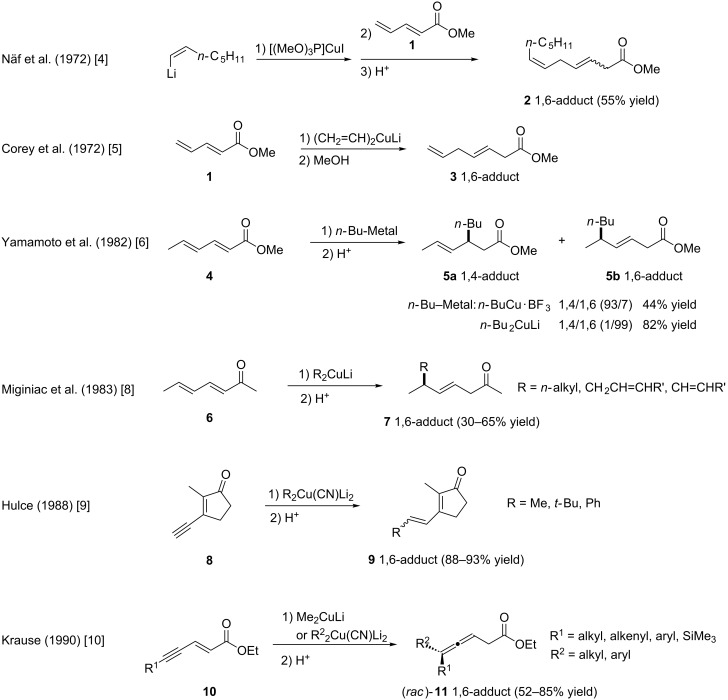
Early reports of conjugate addition of copper-based reagents to extended Michael acceptors.

The first example of achiral addition of a copper-based compound to an extended Michael acceptor was reported independently as early as 1972, by the Näf [4] and Corey [5] groups, who studied the reactivity of pentadienyl methyl ester (**1**, Figure 2). In both cases, the 1,6-conjugate addition of a stoichiometric amount of a Gilman reagent proceeded in a selective manner, affording compounds **2** and **3**. In the early 1980s, Yamamoto and co-workers also studied the reactivity of extended Michael acceptors with regard to the nature of the cuprate reagent; methyl sorbate (**4**) was chosen as a model substrate [6]. This work evidenced that a control of the regioselectivity of the reaction could be achieved with a careful choice of the copper-based nucleophile. Indeed, Yamamoto’s cuprate (*n-*BuCu·BF_3_) led to the 1,4-addition product **5a**, while the 1,6-adduct **5b** was selectively obtained upon reaction with a Gilman reagent. Inspired by these seminal studies, the addition of cuprates was investigated onto different Michael acceptors [7]. The reaction of dienones such as **6** (Miginiac) [8], enynones of the type **8** (Hulce) [9] or polarized enynes **10** (Krause) [10] consistently proceeded with a 1,6-selectivity, as compounds **7**, **9** and **11** were respectively identified as the major reaction product. The selective 1,6-addition of cuprates onto extended Michael acceptors featuring a terminal C–C triple bond prompted research groups to investigate thoroughly the mechanism of this reaction [11–13].

**Figure 3:**
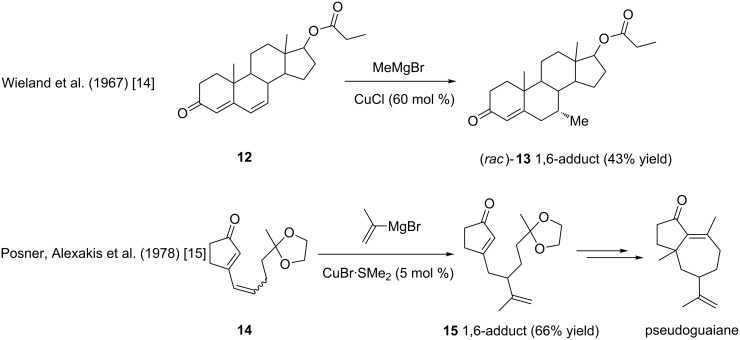
First applications of copper catalyzed 1,6-ACA in total synthesis.

Notably, the 1,6-conjugate additions onto Michael acceptors involving copper reagents were employed in total synthesis strategies (Figure 3). Wieland and Anner took advantage of the reaction selectivity in the synthesis of steroids as early as 1967 [14]. For instance, the product (*rac*)-**13** was obtained in 43% yield by reacting a methylmagnesium bromide with the steroid derivative **12** in the presence of a substoichiometric amount of copper chloride. Ten years later, Alexakis and Posner described the addition of a vinyl Grignard reagent to the conjugated dienone **14**, affording product **15** in 66% yield, ultimately leading to pseudoguaiane [15].

The initial results regarding stoichiometric reactions of copper-based nucleophiles onto extended Michael acceptors gave the scientific community a glimpse of the great potential of such methodologies. It appeared that the regioselectivity of the reaction could be tuned by varying the nature of the copper reagent [6]. Additionally, the applications in total synthesis demonstrated that the nucleophilic copper compound could be generated in situ [14–15]. The design of efficient catalytic protocols could therefore be envisioned, enabling fine-tuning of the regio- and the enantioselectivity of the reaction. In order to tackle this challenge, many research groups extensively investigated the effect of various copper precursors, nucleophiles and Michael acceptors in catalysis, in combination with new families of chiral ligands. The results will be presented according to the selectivity of the conjugate addition; the first section will be dealing with enantioselective 1,6-additions, followed by the description of systems affording preferentially the 1,4-adduct, and a final paragraph will focus on the reactions conditions leading to 1,8- or 1,10-addition products.

### Enantioselective 1,6-addition to extended Michael acceptors

#### With dialkylzinc reagents

**Scheme 1:**
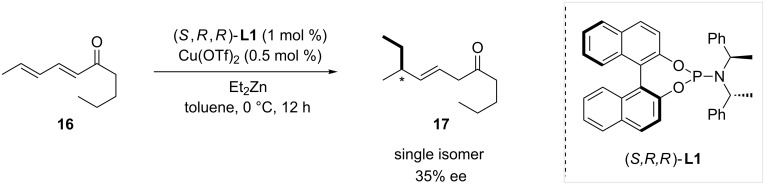
First example of enantioselective copper-catalyzed ACA on an extended Michael acceptor.

Alexakis and co-workers discovered in 2001 the first example of copper-catalyzed enantioselective 1,6-conjugate addition [16]. Using phosphoramidite ligand (*S,R,R*)-**L1** and Cu(OTf)_2_ as the copper source, diethylzinc was added to dienone **16** with a full 1,6-regioselectivity, and an ee of 35% (Scheme 1).

**Scheme 2:**
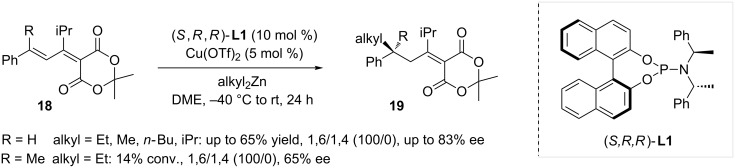
Meldrum’s acid derivatives as substrates in enantioselective ACA.

In 2006, Fillion and co-workers studied the reactivity of Meldrum’s acid derivatives of the type **18** with regards to ACA reactions, using dialkylzinc as nucleophiles [17]. Employing the same catalytic system as Alexakis, namely Cu(OTf)_2_/(*S,R,R*)-**L1**, the reaction was also fully 1,6-selective, and its versatility was studied on a substrate scope. As shown in Scheme 2, tertiary and quaternary stereogenic centers could be generated using this methodology leading to products **19** in moderate to good yields and ees.

**Scheme 3:**
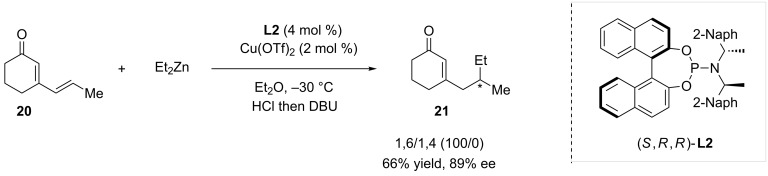
Reactivity of a cyclic dienone in Cu-catalyzed ACA of diethylzinc.

In 2008, Alexakis and Mauduit evaluated a series of different chiral ligands in ACA reactions involving polyenic Michael acceptors and various nucleophiles [18]. In this study, the addition of diethylzinc was notably investigated on cyclic dienone **20** (Scheme 3). As regards the 1,6-conjugate addition, the highest enantioselectivity was achieved with the bulky phosphoramidite (*S,R,R*)-**L2**, to afford **21** in 66% yield and 89% ee.

**Scheme 4:**
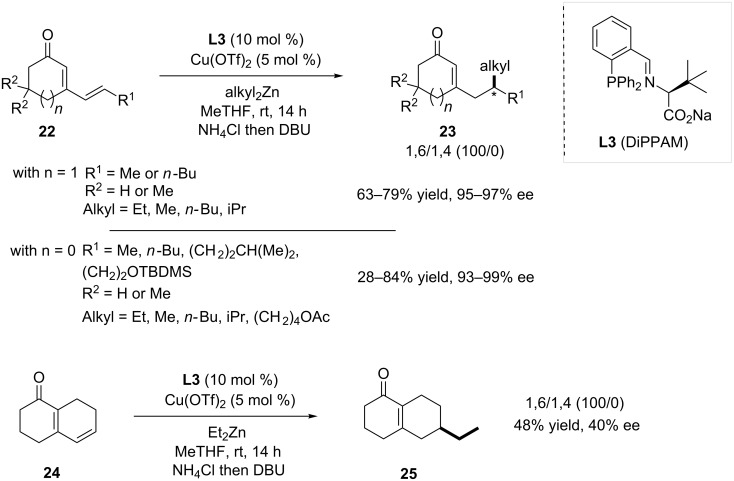
Efficiency of DiPPAM ligand in 1,6-ACA of dialkylzinc to cyclic dienones.

Shortly after, Alexakis and Mauduit demonstrated the efficiency of the carboxylate-phosphino Schiff-base ligand DiPPAM (**L3**) in copper-catalyzed 1,6-ACA with cyclic dienones [19]. Interestingly, the reaction remained fully 1,6-regioselective, while the enantioselectivity was significantly improved. Indeed, a wide variety of substrates of the type **22** were reacted with several dialkylzinc reagents, affording the 1,6-adducts **23** with ees ranging from 93 to 99% (Scheme 4). Moreover, the reactivity of bicyclic dienone **24** was studied in these conditions, but a substantially lower enantioselectivity was recorded (**25** formed in a 40% ee). An additional study dealing with the Cu/DiPPAM-based system in the 1,6-addition demonstrated remarkable nonlinear effects (NLE) [20], which could also be observed in 1,4-ACA on both cyclic and acyclic enones.

**Scheme 5:**
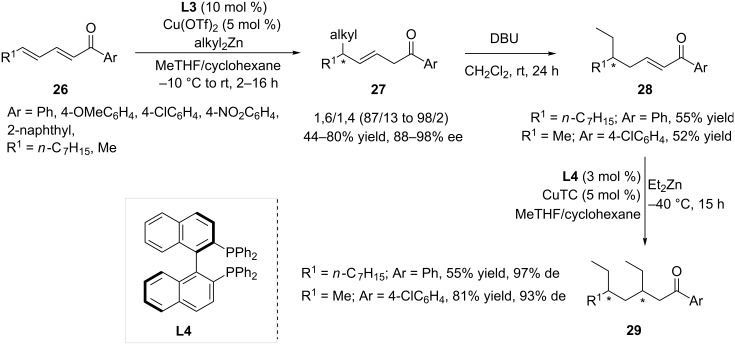
Sequential 1,6/1,4-ACA reactions involving linear aryldienones.

The efficiency of this copper-based catalytic system featuring DiPPAM was further tested in the reaction of linear aryldienones **26**, which are known to be significantly less reactive than their cyclic counterparts [21]. The recorded performances were also excellent, as regioselectivities up to 98/2 and enantioselectivities ranging from 88 to 98% ee were reported. In order to fully demonstrate the synthetic significance of such a methodology, compounds **27** were reconjugated in the presence of DBU and subsequently reacted in the 1,4-ACA (Scheme 5). The optimized conditions for the conversion of **28** to **29** involved copper(I) thiophene-2-carboxylate (CuTC) and (*R*)-Binap **L4,** which afforded the desired final products bearing two stereogenic centers with excellent diastereoselectivies (93–97%).

**Scheme 6:**
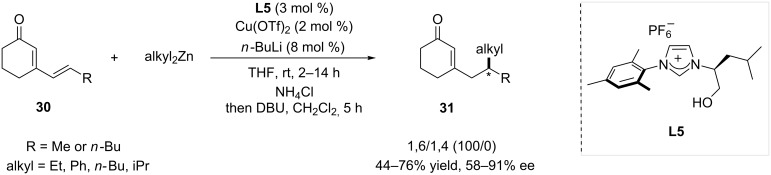
Unsymmetrical hydroxyalkyl NHC ligands in 1,6-ACA of cyclic dienones.

N-Heterocyclic carbenes (NHCs) have emerged, in these last two decades, as a powerful and versatile class of ligands, and appeared to be potent in many catalytic applications [22–23]. Amongst the myriad of available NHC ligands, chiral unsymmetrical NHC ligands appeared as particularly potent in asymmetric catalysis, and were investigated in copper-catalyzed conjugate additions [24]. Recently, a multicomponent synthesis enabled the facile access to a wide variety of unsymmetrical NHC precursors [25]. With this new methodology in hand, Mauduit and co-workers synthesized several bidentate chiral NHC precursors, using amino acids and amino alcohols as starting materials, and tested them in copper-catalyzed ACA [26]. Leucine-based **L5** displayed the best performance in terms of enantioselectivity, and was used in combination with Cu(OTf)_2_ in the 1,6-ACA of cyclic dienones of the type **30** (Scheme 6). NHC ligands also enabled a total regioselectivity and ees ranging from 58 to 91%.

**Scheme 7:**
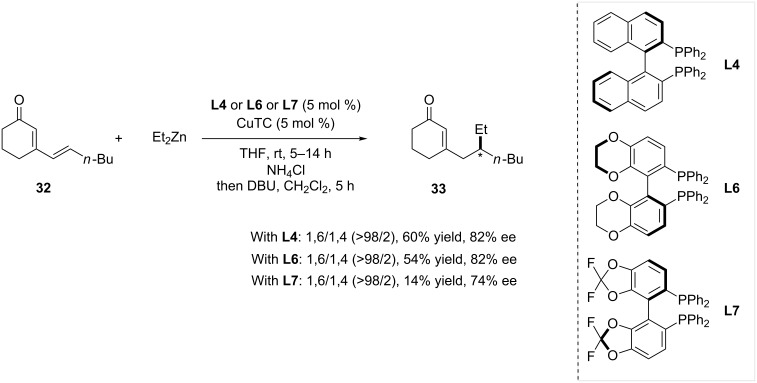
Performance of atropoisomeric diphosphines in 1,6-ACA of Et_2_Zn on cyclic dienones.

Given the efficiency of (*R*)-Binap **L4** in Cu-catalyzed 1,4-ACA on α,β-unsaturated ketones [21], the potency of other atropoisomeric diphosphines was also studied in 1,4 and 1,6-conjugate additions with cyclic and linear substrates [27]. (*S*)-Synphos **L6** and (*R*)-Fluorophos **L7** were used in combination with CuTC and compared to **L4** (Scheme 7). Notably, the reaction of cyclic dienone **32** with diethylzinc proceeded at a typical catalyst loading of 5 mol %, and afforded the 1,6-adduct. Among the ligand series, **L4** proved to form the most efficient system, affording product **33** in 60% yield and 82% ee*.*

#### With Grignard reagents

**Scheme 8:**
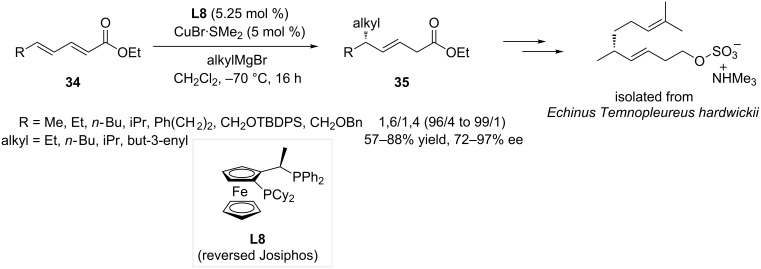
Selective 1,6-ACA of Grignard reagents to acyclic dienoates, application in total synthesis.

Linear dienoates are another class of highly challenging extended Michael acceptors, which were the focus of a work reported by Feringa and co-workers in 2008 [28]. In this study, the efficiency of ferrocene-based ligands was investigated for the addition of Grignard reagents onto ethyl sorbate, using CuBr·SMe_2_ as the copper source. Reversed Josiphos **L8** was selected as the most efficient ligand for this transformation, as a 1,6- vs 1,4-selectivity up to 99:1 could be achieved. The versatility of the catalytic system was assessed with a wide substrate scope featuring aliphatic, aromatic and functionalized dienoates **34** and various Grignard reagents. The reported products **35** were obtained in good yields (57–88%) and excellent ees (72–97%). The applicability of the method was demonstrated with the synthesis of a naturally occurring sulfated alkene, originally isolated from the echinus *Temnopleureus hardwickii* (Scheme 8).

**Scheme 9:**
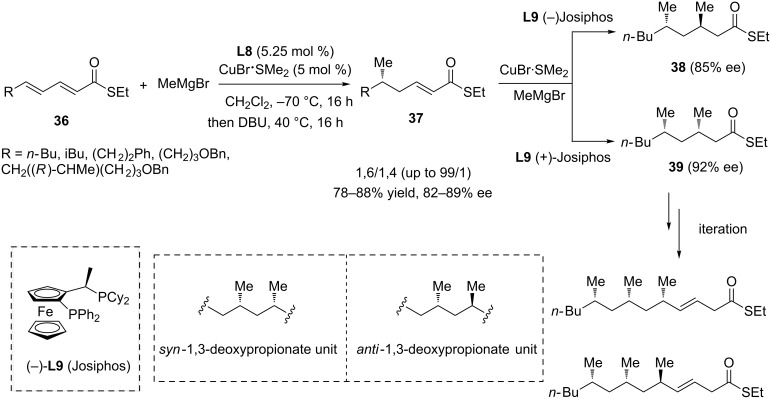
Reactivity of polyenic linear thioesters towards sequential 1,6-ACA/reconjugation/1,4-ACA and production of 1,3-deoxypropionate units.

To be noted, the latter protocol proved unsuccessful in introducing a methyl group in extended Michael acceptors through the addition of MeMgBr. A follow-up study then aimed to tackle this challenge and demonstrated that α,β,γ,δ-unsaturated thioesters **36** were the substrates of choice in order to conduct this valuable transformation (Scheme 9) [29]. A variety of α,β,γ,δ-unsaturated thioesters produced from a Horner–Wadsworth–Emmons reagent were submitted to a 1,6-ACA catalyzed by the **L8**/CuBr·SMe_2_ system, followed by a reconjugation reaction in the presence of DBU to selectively afford **37** (ratio between 1,6 and 1,4-ACA products ranged from 85/15 to 99/1) in high yields (78–88%) and enantioselectivities (82–89%). The obtained α,β-unsaturated thioesters **37** were subsequently reacted in a 1,4 copper-catalyzed ACA, using this time **L9** (Josiphos)/CuBr·SMe_2_. This approach enabled the synthesis of *anti* (**38**) or *syn* (**39**) 1,3-deoxypropionate units depending on the Josiphos enantiomer used, in both cases with good enantioselectivities (85–92% ee). Subsequent chain elongation followed by a 1,4-ACA reaction was described and enabled the enantioselective insertion of an additional methyl group.

#### With trialkylaluminium reagents

**Scheme 10:**
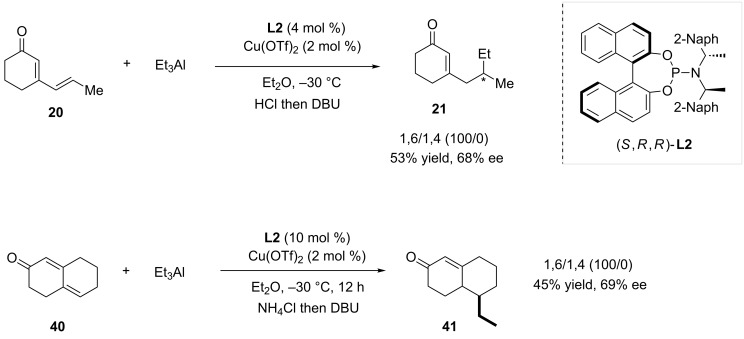
1,6-Conjugate addition of trialkylaluminium with regards to cyclic dienones.

Only a few systems are known to perform efficiently 1,6-ACA reactions using trialkylaluminium reagents as nucleophiles. In 2008, Alexakis, Mauduit and co-workers described the copper-catalyzed 1,6-ACA of triethylaluminium on cyclic α,β,γ,δ-unsaturated ketones using the phosphoramidite (*S,R,R*)-**L2** ligand [18]. The reaction of the cyclic dienone **20** selectively afforded the 1,6-adduct **21** in 53% yield and 68% ee (Scheme 10). Displaying a similar reactivity, bicyclic Michael acceptor **40** led to compound **41** in 45% yield and 69% ee.

**Scheme 11:**
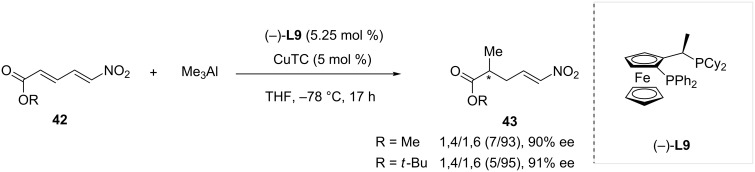
Copper-catalyzed conjugate addition of trimethylaluminium onto nitro dienoates.

In 2010, the Alexakis group explored the reactivity of α,β,γ,δ-unsaturated nitroolefins and nitroenynes in Cu-catalyzed ACA reactions with trialkylaluminium [30]. Several substrates were investigated affording the 1,4-adducts in most cases. However, high 1,6-selectivity with respect to the nitro group could only be observed in the reaction of nitrodienoates **42** with trimethylaluminium (Scheme 11). The most efficient catalytic system, a combination of Josiphos **L9** as chiral ligand and copper thiophene 2-carboxylate (CuTC) afforded the desired 1,6-adducts **43** with very good regioselectivity (up to 5/95) and enantioselectivities (up to 91% ee).

### Enantioselective 1,4-addition to extended Michael acceptors

#### With dialkylzinc reagents

**Scheme 12:**
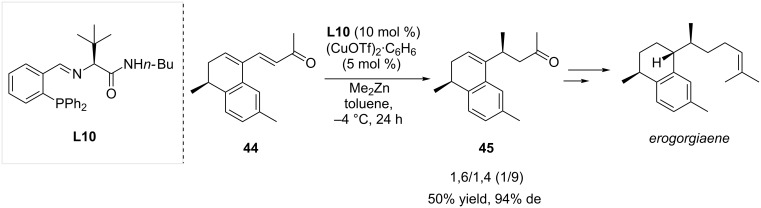
Copper-catalyzed selective 1,4-ACA in total synthesis of erogorgiaene.

Dialkylzinc reagents are without a doubt highly potent nucleophiles in copper-catalyzed ACA often leading to 1,6-adducts onto polyconjugated electron-deficient substrates. However, examples of their use for 1,4-additions can also be found in the literature. In 2004, Hoveyda and co-workers described the total synthesis of the antimicobacterial agent erogorgiaene (Scheme 12) [31]. One of the key steps of this synthesis involved the conversion of the bicyclic extended Michael acceptor **44** to **45** through a 1,4-selective copper-catalyzed ACA. Copper(I) triflate and chiral phosphine ligand **L10** enabled this transformation to proceed with a yield of 50% and an excellent diastereoselectivity (de 94%).

**Scheme 13:**
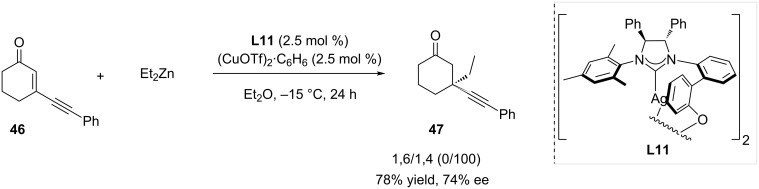
1,4-selective addition of diethylzinc onto a cyclic enynone catalyzed by a chiral NHC-based system.

The Hoveyda group was also interested in developing efficient methods for the generation of quaternary stereogenic centers from Michael acceptors via copper-catalyzed 1,4-ACA of diethylzinc onto cyclic Michael acceptors [32]. When the catalytic system was formed in situ from chiral NHC-based **L11** and (CuOTf)_2_·C_6_H_6_, a large library of substrates was tested, and good yields and ees were consistently observed. Among the Michael acceptors that were submitted to the reaction conditions, cyclic enynone **46** selectively led to the 1,4-adduct **47**, and the ethyl moiety was inserted with 74% enantiomeric excess in 78% yield (Scheme 13).

**Scheme 14:**
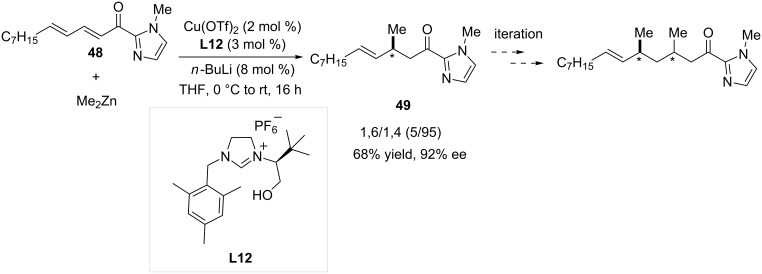
Cu-NHC-catalyzed 1,6-ACA of dimethylzinc onto an α,β,γ,δ-unsaturated acyl-*N*-methylimidazole.

Very recently, a new study dealing with the reactivity of unsaturated acyl-*N*-methylimidazole substrates in copper-catalyzed ACA was released by Mauduit, Campagne and co-workers [33]. Unsymmetrical bidentate hydroxyalkyl precursor **L12** led to the most efficient system in the insertion of methyl groups in such architectures, being highly versatile synthetic platforms [34–35]. A wide variety of α,β-unsaturated acyl-*N*-methylimidazoles could thus be reacted in high yields and enantioselectivities. Notably, the reactivity of polyenic species **48** was also investigated (Scheme 14). Interestingly, the 1,4-adduct was here formed in high regioselectivity (95%), good yield (68%) and stereoselectivity (ee 92%). Interestingly, the usefulness of the products of 1,4-ACA was demonstrated as the latter were converted into the corresponding aldehydes and subsequently used in an iterative process, leading to highly desirable 1,3-deoxypropionate units.

#### With Grignard reagents

**Scheme 15:**
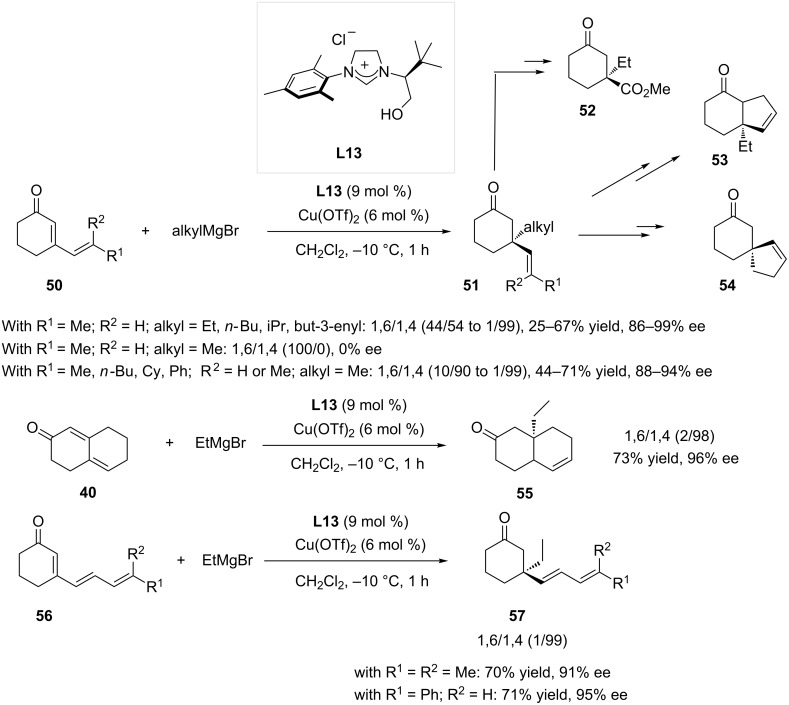
1,4-Selectivity in conjugate addition on extended systems with the concomitant use of a chelating chiral hydroxyalkyl NHC and of a Grignard reagent.

In 2008, Alexakis, Mauduit and coworkers extensively studied the influence of the parameters controlling the regioselectivity outcome of the ACA reaction with α,β,δ,γ-unsaturated ketones [18]. Using a Grignard reagent as the nucleophile, it appeared that catalytic systems based on phosphoramidite ligands favored the formation of the 1,6-adduct. However, the use of catalytic systems based on an hydroxyalkyl NHC ligand (Cu(OTf)_2_/**L13**) resulted in a surprising inversion of the regioselectivity. Indeed, the addition of ethylmagnesium bromide onto cyclic dienones occurred at the 1,4-position, affording compounds featuring an all-carbon quaternary center. The authors suggested that the chelating hydroxyalkyl chain was at the origin of this particular reactivity. The addition of other linear Grignard reagents on the substrates of the type **50** showed a near-perfect 1,4-regioselectivity, while the amount of 1,4-adduct dropped when branched nucleophiles were used. Despite this decrease in regioselectivity, the reaction remained highly enantioselective, with ees ranging from 88 to 99% for compounds **51**. Notably, attempts to add a methyl moiety through the addition of MeMgBr to the cyclic dienone featuring a disubstituted terminal double bond (R^1^ = Me, R^2^ = H) only resulted in an achiral 1,6-addition product. Subsequent transformations of the γ,δ-unsaturated 1,4-adducts were successfully performed: an oxidative cleavage afforded for example ketoester **52** (Scheme 15). Moreover, the in situ trapping of the addition product with acetic anhydride led to the regeneration of the lithium enolate, which was allylated and submitted to ring closing metathesis to afford the bicyclic product **53**. Finally, the RCM of the 1,4-adduct resulting from the addition of 3-butenylmagnesium bromide yielded the spiro compound **54**. Interestingly, the conversion of bicyclic compound **40** catalyzed by the same system also occurred selectively in the 4-position (**55** was formed in 73% yield, 96% ee).

The scope of the reaction was extended to many new substrates in 2012, evidencing that the 1,4-selectivity of the transformation remained with trienones **56**, as the polyunsaturated products **57** were obtained with good yield and excellent enantioselectivities (up to 95%) [36].

**Scheme 16:**
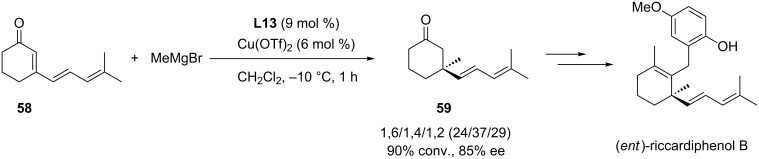
Cu-NHC catalyzed 1,4-ACA as the key step in the total synthesis of *ent*-riccardiphenol B.

To demonstrate the high synthetic significance of a selective 1,4-ACA performed on extended systems, its implementation in the total synthesis of *ent*-riccardiphenol B was attempted using the **L13**/Cu(OTf)_2_ system (Scheme 16) [36]. The conditions for the addition of methylmagnesium bromide were varied in order to maximize the conversion of trienone **58** towards **59**. A mixture of the three addition products was however consistently observed, and despite an ee of 85%, the conversion towards the desired product did not exceed 37%.

**Scheme 17:**
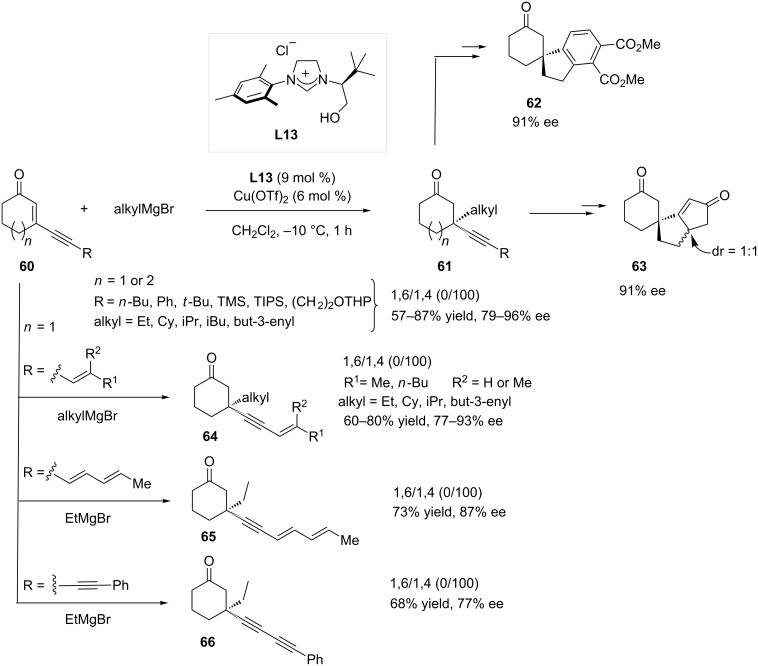
Cu-NHC-catalyzed 1,4-selective ACA reactions with enynones.

Cyclic enynones **60**, which are substrates of high interest for the synthetic chemist, were regioselectively converted in good yields to the 1,4-ACA products **61** with good to excellent enantioselectivity (79 to 96% ee) using the **L13**/Cu(OTf)_2_ catalytic system [36–37]. It is important to note that the regioselectivity outcome for the addition of methyl Grignard reagent appeared to be more substrate-dependent [36]. In fact, the best 1,4-selectivities were observed with bulky R groups, as a 100/0 1,4/1,6 ratio was observed with R = TIPS while substrate **60** with R = *n*-Bu afforded a 23/77 1,4/1,6 ratio. Moreover, the obtained products **61** could be subsequently converted to the spiro compounds **62** and **63** (Scheme 17).

The same protocol was later applied to conjugated enynones featuring additional unsaturated units and a total regioselectivity towards the 1,4-adducts was recorded under the standard conditions (Scheme 17) [36]. The chiral polyconjugated products (**64**–**66**) were isolated in good yields (60 to 80% yields) with good to excellent enantioselectivity (77 to 93% ee).

**Scheme 18:**
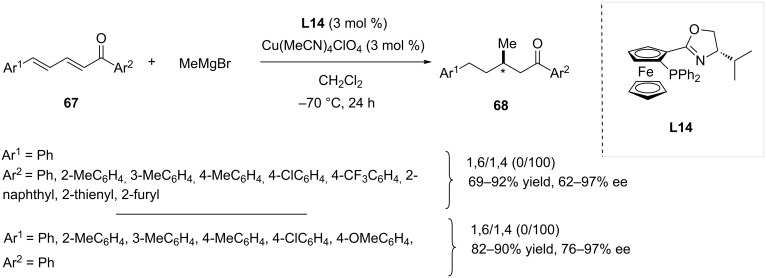
Linear dienones as substrates in 1,4-asymmetric conjugate addition reactions of Grignard reagents catalyzed by a copper-based system.

In 2013, Xie, Zhang and co-workers investigated the reactivity of extended unsaturated linear ketones in the presence of Grignard reagents, with the aim of selectively forming the 1,4-ACA adducts [38]. Various ligands were tested and the best catalytic performance was achieved using bidentate ferrocene-based ligand **L14** in combination with tetrakis(acetonitrile)copper(I) perchlorate as the copper source. The conversion of aromatic linear dienones **67** was reported with a complete regioselectivity towards the 1,4-adducts **68**. Moreover, the variation of the steric and electronic parameters of both aromatic moieties of **67** confirmed the robustness of the method, with good yields and ees obtained in most cases (Scheme 18).

#### With trialkylaluminium reagents

**Scheme 19:**
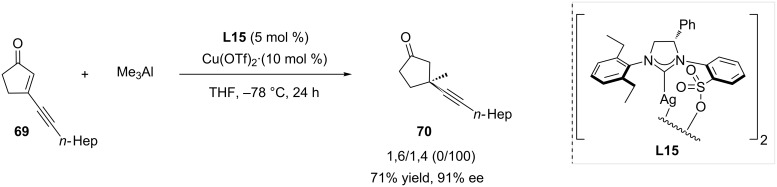
1,4-ACA of trimethylaluminium to a cyclic enynone catalyzed by a copper-NHC system.

The Hoveyda group disclosed the first example of copper-catalyzed selective 1,4-ACA of low-cost trialkylaluminium reagents on extended Michael acceptors in 2008 [39]. The reported catalytic system, featuring Cu(OTf)_2_ and sulfonated NHC-based silver complex **L15** as the ligand source, appeared as the most potent system in the conversion of cyclic enones. Enynone **69** was reacted to assess the versatility of the reaction, and a full 1,4-regioselectivity was recorded, leading to compound **70** in 71% yield and 91% ee (Scheme 19).

**Scheme 20:**
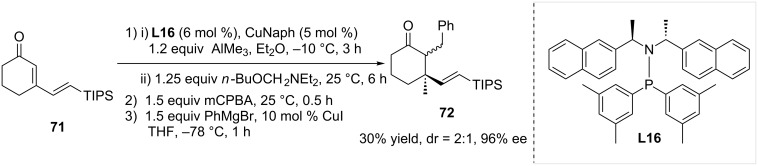
Generation of a sterically encumbered chiral cyclohexanone from a polyunsaturated cyclic Michael acceptor.

Another example of trialkylaluminium addition onto a cyclic extended Michael acceptor was reported in 2013, using a combination of copper(II) naphthenate (CuNaph) and SimplePhos **L16** as the catalytic system [40]. The reported methodology involved a regioselective 1,4 ACA of trimethylaluminium followed by the trapping of the aluminium enolate intermediate with (*n*-butoxymethyl)diethylamine. An oxidation–elimination sequence and a conjugate addition of a Grignard reagent to the newly formed exocyclic double bound were subsequently performed. Overall, this four-step process afforded the sterically congested cyclohexanone **72** in a 30% overall yield, with a dr of 2:1 and 96% ee (Scheme 20).

**Scheme 21:**
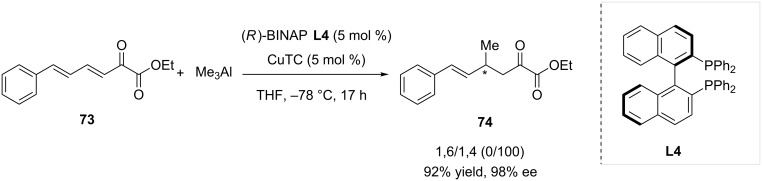
Selective conversion of β,γ-unsaturated α-ketoesters in copper-catalyzed asymmetric conjugate addition.

In 2012, Alexakis and Gremaud studied the reactivity of various β,γ-unsaturated α-ketoesters, which remain to date the only report dealing with such substrates in Cu-catalyzed ACA of trialkylaluminium [41]. Using the (*R*)-Binap-based system **L4**/CuTC, an excellent 1,4-selectivity was achieved with mono-unsaturated substrates. Dienic ketoester **73** was also tested with this catalytic system, and 1,4-adduct **74** was formed with a perfect regioselectivity, in high 92% yield and with a remarkable enantioselectivity of 98% (Scheme 21).

**Scheme 22:**
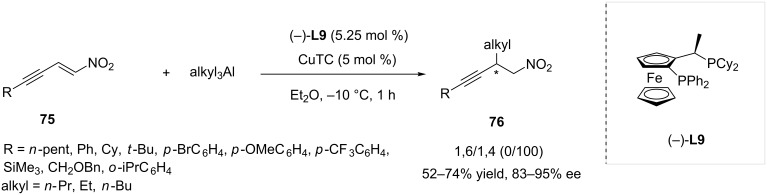
Addition of trialkylaluminium compounds to nitroenynes catalyzed by **L9**/CuTC.

In their report looking into the reactivity of α,β,γ,δ-unsaturated nitroolefins [30], Alexakis and co-workers could also attain a perfect 1,4-regioselectivity in the addition of trialkylaluminium reagents. Many nitroenynes such as **75** could then be converted to a wide variety of enantioenriched products **76** (83 to 95% ee) using copper thiophenecarboxylate (CuTC) as the copper source and Josiphos **L9** as chiral ligand (Scheme 22).

**Scheme 23:**
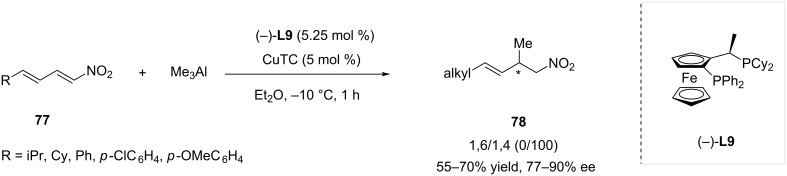
Addition of trialkylaluminium compounds to nitrodienes catalyzed by **L9**/CuTC.

Additionally, a collection of nitrodienes **77** was reacted using this same methodology, and a reactivity similar to the one of nitroenynes was observed: 1,4-adducts **78** were obtained with good to very good enantioselectivity (77 to 90% ee) and perfect regioselectivity (Scheme 23).

### Enantioselective 1,10- and 1,8-addition to extended Michael acceptors

**Scheme 24:**
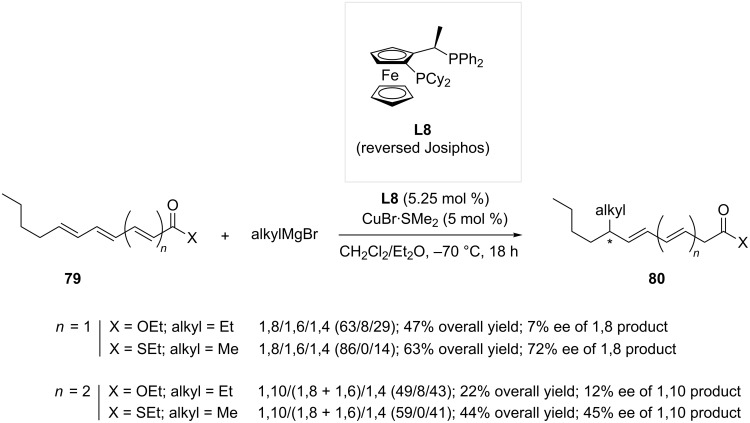
Copper catalyzed 1,8- and 1,10-ACA reactions.

Recently, Feringa and co-workers released an extensive work dealing with the influence of various parameters in copper-catalyzed ACA of Grignard reagents on extended Michael acceptors [42]. Using “reversed Josiphos” **L8**, a well established ligand for enantio- and regioselective 1,6-additions onto dienoates [28], a wide variety of polyconjugated substrates were tested in order to gain a better insight into the reaction mechanism. Amongst the investigated electrophiles, substrates **79** (*n* = 1 or 2) could potentially undergo 1,8- or 1,10-conjugated addition, respectively. As shown in Scheme 24, the addition of the Grignard reagent occurred preferentially at the most remote olefin. To be noted, polyenic esters gave a slight regioselectivity towards the 1,8- and 1,10-products and low enantioselectivities. Remarkably, only a small portion (<10%) of “intermediate” addition products (1,6-adducts when *n* = 1 and 1,6- and 1,8-adducts when *n* = 2) was detected in all cases. The nature of the substrate seemed to also have an influence since better results were obtained when the thioester was used as a starting material, in both 1,8- (63% yield, 72% ee) and 1,10-ACA (44% yield, 45% ee). As a trend, the regio- and stereoselectivity decreased when the reacting olefin is further from the electron-withdrawing functionality. Additional studies would enable to determine the factors allowing for an improvement of the reaction outcome, as an efficient protocol allowing 3 to 4 sequential ACA reactions would be highly desirable for synthetic chemists.

## Conclusion

This review attempts to give the reader an overview of the methodologies available to perform regio- and enantioselective copper-catalyzed asymmetric conjugate additions (ACA) on electron-deficient extended unsaturated systems. Since the initial discoveries looking into the conjugate addition of cuprates to extended Michael acceptors, substantial research has been undertaken to develop efficient methodologies enabling such reactions in a regio- and enantioselective manner, with significant breakthroughs. Nowadays, a number of ACA procedures with a various electron-deficient polyenic substrates (linear and cyclic dienones, dienoates, conjugated thioesters, nitroolefins and nitroenynes, enynones, unsaturated acyl-*N*-methylimidazoles and conjugated ketoesters) and nucleophiles (dialkylzinc, Grignard or trialkylaluminium reagents) are available. These protocols have shown to be highly dependent on both substrates and reaction conditions. Therefore, a variety of efficient chiral ligands is now available for the chemist willing to design synthetic routes leading to complex chiral molecules. Nonetheless, a number of potential Michael acceptors and catalytic systems have yet to be explored in order to further expand this useful toolbox.
